# BMC Cardiovascular Disorders reviewer acknowledgement 2014

**DOI:** 10.1186/1471-2261-15-2

**Published:** 2015-04-07

**Authors:** Tim Shipley

**Affiliations:** BioMed Central, Floor 6, 236 Gray’s Inn Road, London, WC1X 8HB United Kingdom

## Abstract

The editors of BMC Cardiovascular Disorders would like to thank all our reviewers who have contributed to the journal in Volume 14 (2014).

**Pedro Abizanda**

Spain

**Vikram Agarwal**

United States of America

**Umme Aiman**

India

**Muharrem Akin**

Germany

**Majd Al Ghatrif**

United States of America

**Stephane Allouche**

France

**Abdallah Al-Mohammad**

United Kingdom

**Alvaro Alonso**

United States of America

**Bahaaldin Alsoufi**

United States of America

**Rachel Amiya**

Japan

**Enrico Ammirati**

Italy

**Daraban Ana Maria**

Romania

**Ajith Ananthakrishna Pillai**

India

**Annalisa Angelini**

Italy

**Emiliano Angeloni**

Italy

**Uzair Ansari**

Germany

**Pamella Anunciação**

Brazil

**Ashraf Anwar**

Saudi Arabia

**Yaron Arbel**

Israel

**Carmelina Ariano**

Italy

**Rakesh Arora**

Canada

**Dharamvir Arya**

India

**Kei Asayama**

Belgium

**Ajax Atta**

Brazil

**Wael Attia**

Egypt

**Johann Auer**

Austria

**Anna Axelsson**

Denmark

**Flavia Ballocca**

Italy

**Sevket Balta**

Turkey

**Frédéric Banal**

France

**Marco Barbanti**

Italy

**Umberto Barbero**

Italy

**Maria Barcelo**

Spain

**Fellype Barreto**

Brazil

**Pier Paolo Bassareo**

Italy

**Rob Bauer**

United States of America

**Chirag Bavishi**

United States of America

**Alexander Becker**

Germany

**Hassan Behlouli**

Canada

**Michael Behnes**

Germany

**Iddo Ben-Dov**

Israel

**Kálmán Benke**

Hungary

**Knut Erik Berge**

Norway

**Alessandro Bernardi**

Italy

**Ishir Bhan**

United States of America

**Kartikeya Bhargava**

India

**Jacek Bialkowski**

Poland

**Matteo Bianco**

Italy

**Mandy Binning**

United States of America

**Federico Bizzarri**

Italy

**Emanuela Bostjancic**

Slovenia

**Marouane Boukhris**

Tunisia

**Marc Buijsrogge**

Netherlands

**Alida Caforio**

Italy

**Adriano Caixeta**

Brazil

**Gianluca Campo**

Italy

**Ahmet Selçuk Can**

Turkey

**Xu Cao**

United States of America

**Marina Cerbo**

Italy

**John Chambers**

United Kingdom

**Steven Chamuleau**

Netherlands

**Hoi Chan**

United States of America

**Po-Yuan Chang**

Taiwan

**Meng-Hua Chen**

China

**Shaojie Chen**

China

**Hsin-Jen Chen**

United States of America

**Xiangmei Chen**

China

**Benjamin Cheong**

United States of America

**Jae Hyung Cho**

United States of America

**Rocky Chow**

Taiwan

**Imke Christiaans**

Netherlands

**Thomas Christophersen**

Denmark

**Pao-Hsien Chu**

Taiwan

**Siew Siang Chua**

Malaysia

**Shao-Yuan Chuang**

Taiwan

**Antonio Ciccarelli**

Italy

**Alexander Clark**

Canada

**Aldo Clerico**

Italy

**Sean Coady**

United States of America

**Steven Coca**

United States of America

**Dennis Cokkinos**

Greece

**Federico Conrotto**

Italy

**Adolfo Correa**

United States of America

**Xenophon Costeas**

Greece

**Antonio Crisafulli**

Italy

**Agnieszka Cudnoch-Jedrzejewska**

Poland

**Douglas Curran-Everett**

United States of America

**Cesare Cuspidi**

Italy

**Saleem Dabbah**

Israel

**Hiroyuki Daida**

Japan

**Fabrizio D'Ascenzo**

Italy

**Linda De Heer**

Netherlands

**Wouter De Ruijter**

Netherlands

**Renato De Vecchis**

Italy

**Kevin Deans**

United Kingdom

**Michael Debney**

United Kingdom

**Jean-Marie Degryse**

Belgium

**Victoria Delgado**

Netherlands

**Christian Delles**

United Kingdom

**Yvan Devaux**

Luxembourg

**Permphan Dharmasaroja**

Thailand

**Giuseppina Di Biase**

Italy

**Giovanni Di Salvo**

Italy

**Frank Dini**

Italy

**James Di Nicolantonio**

United States of America

**Torsten Doenst**

Germany

**Rudin Domi**

Albania

**Chiara Donfrancesco**

Italy

**Mei Dong**

China

**Rami Doukky**

United States of America

**Danny Dvir**

Canada

**Nicole Ebner**

Germany

**Elisa Ebrille**

Italy

**Ulf Ekelund**

Sweden

**John Elefteriades**

United States of America

**Hazem Elewa**

United States of America

**Salma Esmaeil**

Egypt

**Rodrigo Estevez-Loureiro**

Spain

**Leanne Felkin**

United Kingdom

**Timothy Fendler**

United States of America

**Ignacio Ferreira**

Spain

**Claudia Fiorina**

Italy

**Sara Fitzgerald-Butt**

United States of America

**Joost Fledderus**

Netherlands

**Sebastien Foulquier**

Netherlands

**Marcus Franz**

Germany

**Marie Freel**

United Kingdom

**Lars Frost**

Denmark

**Yoshihiro Fukumoto**

Japan

**Adelaide Fusco**

Italy

**Angelo Gaffo**

United States of America

**Ujjawal Gandhi**

United States of America

**Ron Gansevoort**

Netherlands

**Lei Gao**

China

**Carlo Gaudio**

Italy

**J. Christoph Geller**

Germany

**Jeffrey Geske**

United States of America

**Sebastiano Gili**

Italy

**Thomas Gillette**

United States of America

**Francesca Giordana**

Italy

**Grégoire Girod**

Switzerland

**Stephen Glasser**

United States of America

**Tadhg Gleeson**

Ireland

**Sameer Goyal**

India

**Anders M. Greve**

Denmark

**Eric Gross**

United States of America

**Wan-Jie Gu**

China

**Andrey Gudkov**

Russian Federation

**Hartmut Karl Guelker**

Germany

**Hervé Guillou**

France

**Oliver Gunkel**

Germany

**Mark Haigney**

United States of America

**Nobuyuki Hamajima**

Japan

**Job Harenberg**

Germany

**David Haslam**

United Kingdom

**Eleni Hatzinikolaou-Kotsakou**

Greece

**Jan Heeringa**

Netherlands

**Kevin Heffernan**

United States of America

**Martin Holzmann**

Sweden

**Yuling Hong**

United States of America

**Dongming Hou**

United States of America

**Sandy Hu**

China

**Po Hsun Huang**

Taiwan

**Frank Hulstaert**

Belgium

**Holger Husi**

United Kingdom

**Olga Husson**

Netherlands

**Mario Iannaccone**

Italy

**Motoyuki Iemitsu**

Japan

**Massimo Imazio**

Italy

**Hart Isaacs**

United States of America

**Yasushi Ishigaki**

Japan

**Yoshitaka Iwanaga**

Japan

**Raffaele Izzo**

Italy

**Frederic Jacques**

Canada

**Sae Young Jae**

Korea, South

**Piia Jallinoja**

Finland

**Ruben Jara-Rubio**

Spain

**Li Jia**

China

**Edward Johns**

Ireland

**Alfonso Jurado-Roman**

Spain

**Edo Kaluski**

United States of America

**Petra Kameritsch**

Germany

**Bharat Kantharia**

United States of America

**Mehran Karimi**

Iran

**Mahwash Kassi**

United States of America

**Hisashi Kawashima**

Japan

**Do Han Kim**

Korea, South

**Ajay Kirtane**

United States of America

**Soichiro Kitamura**

Japan

**Jesper Kjaergaard**

Denmark

**Julie Klein**

France

**Peter Klein-Weigel**

Germany

**Gert Klug**

Austria

**Tijmen Koëter** Netherlands

**Andrew Kompa**

Australia

**Deling Kong**

China

**Alexandra Konradi**

Russian Federation

**David Koolbergen**

Netherlands

**Jelena Kornej**

Germany

**Johannes Kragten**

Netherlands

**Martin Krakauer**

Denmark

**Mahendra Kumar**

United States of America

**Dharam Kumbhani** United States of America

**Sung Uk Kwon**

Korea, South

**Michael La Fountaine**

United States of America

**Chun-Fu Lai**

Taiwan

**Malinee Laopaiboon**

Thailand

**Camille Lassale**

United Kingdom

**Johan Lassus**

Finland

**Crystal Lee**

Australia

**Won Young Lee**

Korea, South

**Ju-Mi Lee**

Korea, South

**Jung Eun Lee**

Korea, South

**Yau-Jiunn Lee**

Taiwan

**Gilles Lemesle**

France

**Clive Lewis**

United Kingdom

**Hung-Yuan Li**

Taiwan

**Clare Liddy**

Canada

**Edgars Liepins**

Latvia

**Lian-Yu Lin**

Taiwan

**Lisa Lincz**

Australia

**Daniel Lingamfelter**

United States of America

**Eric Lopatta**

Germany

**Oscar Lorenzo**

Spain

**Eric Loucks**

United States of America

**Nicole Lowres**

Australia

**Xinwu Lu**

China

**Xiaofang Lu**

United States of America

**Andrew Ludman**

United Kingdom

**Joanne Luke**

Australia

**David Maciver**

United Kingdom

**Judith Mackall**

United States of America

**Jared Magnani**

United States of America

**Ahmed Mahmoud**

United States of America

**Bernhard Maisch**

Germany

**Olebogeng Majane**

South Africa

**Bilal Majed**

France

**Shinji Makita**

Japan

**Boris A. Malyarchuk**

Russian Federation

**Nassos Manginas**

Greece

**Samer Mansour**

Canada

**Luis Manzano**

Spain

**Themistoklis Maounis**

Greece

**Germanas Marinskis**

Lithuania

**Patrick Mark**

United Kingdom

**Pedro Marques Da Silva**

Portugal

**Ruth Masterson Creber**

United States of America

**Guido A. Matschuck**

Germany

**Sophie Mavrogeni**

Greece

**Otto Mayer**

Czech Republic

**Agnes Mayr**

Austria

**Terence Mccann**

Australia

**Alex Mclellan**

Australia

**Nehal Mehta**

United States of America

**Puja Mehta**

United States of America

**Catharina Mels**

South Africa

**Jort Merken**

Netherlands

**Bernhard Metzler**

Austria

**Sven Meyer**

Germany

**Thomas Meyer**

Germany

**Antonio Miceli**

Italy

**Akiko Mii**

Japan

**Alma Mingels**

Netherlands

**Yoko Miyasaka**

Japan

**Pietro Amedeo Modesti**

Italy

**Misbahuddin Mohammad**

United Kingdom

**Francois-Pierre Mongeon**

Canada

**Nuno Moreno**

Portugal

**I Haldun Müderrisoglu**

Turkey

**Christian Mueller**

Switzerland

**Hans-Wilhelm Müller**

Germany

**Venkatesh Murthy**

United States of America

**Karthik Murugiah**

United States of America

**Hazumu Nagata**

Japan

**Maria Navas**

United States of America

**Isao Nishi**

Japan

**Marko Noc**

Slovenia

**Youssef Nosir**

Saudi Arabia

**Ntobeko Ntusi**

United Kingdom

**Evangelia Ntzani**

Greece

**Gaetano Nucifora**

Italy

**Naro Ohashi**

Japan

**Sadanori Okada**

Japan

**Takanobu Okamoto**

Japan

**Elena Okisheva**

Russian Federation

**Takafumi Okura**

Japan

**Altan Onat**

Turkey

**Michelle Orme**

United Kingdom

**Christian Ott**

Germany

**Linda Oude Griep**

United Kingdom

**Sara Palacio Restrepo**

Italy

**Barry Palmer**

New Zealand

**Matthew Panagiotou**

Greece

**Anil Pandit**

United States of America

**Constantinos Pantos**

Greece

**Guido Parodi**

Italy

**Jeetesh Patel**

United Kingdom

**Matthias Pauschinger**

Germany

**Marco Pavani**

Italy

**Charles Pearman**

United Kingdom

**Dudley Pennell**

United Kingdom

**Rebecca Perry**

Australia

**Pawel Petkow Dimitrow**

Poland

**Elisa Picardi**

Italy

**Claudio Picariello**

Italy

**Stefano Pidello**

Italy

**Anna Pilbrow**

New Zealand

**Bertram Pitt**

United States of America

**Carmine Pizzi**

Italy

**Alberto Polimeni**

Italy

**Katrina Poppe**

New Zealand

**Janet Powell**

United Kingdom

**Jeppe Praetorius**

Denmark

**David Preiss**

United Kingdom

**Riccardo Proietti**

Italy

**Armando Pucciarelli**

Italy

**Rajesh Puranik**

Australia

**Rishi Puri**

Canada

**Giorgio Quadri**

Italy

**Alicia Quirós**

Spain

**Amresh Raina**

United States of America

**Sushant Ranadive**

United States of America

**Eleni Rapsomaniki**

United Kingdom

**Brian Rayner**

South Africa

**David Rehkopf**

United States of America

**Giuseppe Rengo**

Italy

**Paul Rheeder**

South Africa

**Angelos Rigopoulos**

Germany

**Guillermo Rodrigo**

Spain

**Isabel Rodriguez**

Spain

**Bruno Roseguini**

Brazil

**Francesco Rotondi**

Italy

**Santiago Roura**

Spain

**Jason Roy**

United States of America

**Erdal Safak**

Germany

**Samir Saha**

Sweden

**Tayfun Sahin**

Turkey

**Gianmario Sambuceti**

Italy

**Ana Santos**

Portugal

**Kátia Santos**

Brazil

**Nizal Sarrafzadegan**

Iran

**Filippo Sarullo**

Italy

**Hiroyuki Sasai**

United States of America

**Patrick Savage**

United States of America

**Ana Savic-Radojevic**

Serbia

**Hartzell Schaff**

United States of America

**Bruno Scheller**

Germany

**Francois Schellevis**

Netherlands

**Thomas Schindler**

United States of America

**Markus Schneider**

Germany

**Andreas Schoenenberger**

Switzerland

**Paul Schoof**

Netherlands

**Mikkel Malby Schoos**

Denmark

**Barbara Schumann**

Sweden

**Gregory Schwartz**

United States of America

**Robert Scragg**

New Zealand

**David Segal**

Australia

**Jitka Seidlerová**

Czech Republic

**Christine Seidman**

United States of America

**Gregory Sgueglia**

Italy

**Ryan Sheldon**

United States of America

**Motohiro Shimizu**

Japan

**Toshiharu Shinoka**

United States of America

**Supriya Shore**

United States of America

**Mohamed Shoukri**

Saudi Arabia

**Curt Sigmund**

United States of America

**Isaac Silva**

Brazil

**Mauro Silvestrini**

Italy

**Andrew Sindone**

Australia

**Mukesh Singh**

United States of America

**Asru Kumar Sinha**

India

**Neil Smart**

Australia

**Wayne Smith**

South Africa

**Michelle Snyder**

United States of America

**Yoshimitsu Soga**

Japan

**Steinar Solberg**

Norway

**Hong Ji Song**

Korea, South

**Alexandra Sousa**

Portugal

**Adrian Specogna**

Canada

**Anthony Staines**

Ireland

**George Stavridis**

Greece

**Giulio Stefanini**

Switzerland

**Pierluigi Stefano**

Italy

**Paul Stein**

United States of America

**Louis-Mathieu Stevens**

Canada

**Welma Stonehouse**

New Zealand

**Stefan Stortecky**

Switzerland

**Richard Sutton**

Monaco

**Marijana Tadic**

Serbia

**Genzou Takemura**

Japan

**Yodo Tamaki**

Japan

**Weihong Tang**

United States of America

**Manabu Taniguchi**

Japan

**Bamidele O. Tayo**

United States of America

**Antonio Tello-Montoliu**

Spain

**Martin Teraa**

Netherlands

**Sermin Tetik**

Turkey

**Randall Thompson**

United States of America

**Jakob Hartvig Thomsen**

Denmark

**Rebecca Thornhill**

Canada

**Jingwei Tian**

China

**Yaping Tian**

China

**Kimimasa Tobita**

United States of America

**Giuliano Tocci**

Italy

**Salvatore Tomasello**

Italy

**Per Tornvall**

Sweden

**Richard Toshner**

United Kingdom

**Lavinia Tran**

Australia

**Danijela Trifunovic**

Serbia

**Dimitrios Tsiapras**

Greece

**Karam Turk-Adawi**

United States of America

**Dimitrios Tziakas**

Greece

**Ioanna Tzoulaki**

Greece

**Anetta Undas**

Poland

**Roland Van Kimmenade**

Netherlands

**Johannes Van Rooyen**

South Africa

**Niels Van Royen**

Netherlands

**Moniek Van Zitteren**

Netherlands

**Praveen Veerabhadrappa**

United States of America

**Alessandro Verde**

Italy

**Richard Verrier**

United States of America

**Isabelle Vivodtzev**

France

**Veronica Ines Volberg**

Argentina

**Clemens Von Schacky**

Germany

**Franz Von Ziegler**

Germany

**Carina Wallgren-Pettersson**

Finland

**Haixia Wang**

United States of America

**Daowen Wang**

China

**Mingyi Wang**

United States of America

**Tzung-Dau Wang**

Taiwan

**Ralf Wassmuth**

Germany

**Lewis Watson**

United States of America

**Heinz Weber**

Austria

**Bin Wei**

United States of America

**Lei Wei**

United States of America

**Niels Wiinberg**

Denmark

**Lars Wiklund**

Sweden

**Robert Wojciechowski**

United States of America

**Elizabeth Woodcock**

Australia

**Zonggui Wu**

China

**Ameeta Yaksh**

Netherlands

**Tomohide Yamada**

Japan

**Chih-Yu Yang**

Taiwan

**Yuichiro Yano**

United States of America

**Roseanne Yeung**

Canada

**Ali Yilmaz**

Germany

**Kai Hang Yiu**

Hong Kong

**Shi-Min Yuan**

China

**Kyeong Ho Yun**

Korea, South

**Katherine Yutzey**

United States of America

**Xinbo Zhang**

United States of America

**Luxia Zhang**

China

**Yuntao Zhao**

China

**Yinjun Zhao**

United States of America

**Stefan Zimmermann**

Germany

